# High-Density Cobalt Nanoparticles Encapsulated with Nitrogen-Doped Carbon Nanoshells as a Bifunctional Catalyst for Rechargeable Zinc-Air Battery

**DOI:** 10.3390/ma12020243

**Published:** 2019-01-12

**Authors:** Shuqi Liang, Ce Liang

**Affiliations:** Key Laboratory of Automobile Materials, Ministry of Education, and College of Materials Science and Engineering, Jilin University, Changchun 130025, China; lsqjlu@163.com

**Keywords:** cobalt nanoparticles, nitrogen-doped carbon nanoshell, ORR, OER, zinc-air battery

## Abstract

High efficient electrocatalytic activity and strong stability to both oxygen reduction reaction (ORR) and oxygen evolution (OER) are very critical to rechargeable Zn-air battery and other renewable energy technologies. As a class of promising catalysts, the nanocoposites of transition metal nanoparticles that are encapsulated with nitrogen-doped carbon nanoshells are considered as promising substitutes to expensive precious metal based catalysts. In this work, we demonstrate the successful preparation of high-density cobalt nanoparticles encapsulated in very thin N-doped carbon nanoshells by the pyrolysis of solid state cyclen-Co-dicyandiamide complex. The morphologies and properties of products can be conveniently tuned by adjusting the pyrolysis temperature. Owing to the synergetic effect of hybrid nanostructure, the optimized Co@N-C-800 sample possesses outstanding bifunctional activity for both ORR and OER in alkaline electrolyte. Meanwhile, the corresponding rechargeable zinc-air battery that is based on Co@N-C-800 air cathode also has excellent current density, low charge-discharge voltage gap, high power density, and strong cycle stability, making it a suitable alternative to take the place of precious metal catalysts for practical utilization.

## 1. Introduction

The growing environmental degradation and the increasing requirements for renewable energy have stimulated great interest in exploring sustainable energy storage and conversion devices from hydrogen production by water splitting to metal-air battery [1]. Among various energy storage and conversion devices, zinc-air battery has been regarded as one of the promising candidates for the electrical vehicles (EVs), owing to its low-cost, high safety, and high energy density [2,3,4,5]. However, the electrochemical properties of rechargeable Zn-air battery are still far from large-scale applications due to the limitation of oxygen electrode, which is suffering from not only the large overpotential resulting from the sluggish kinetics of oxygen reduction reaction (ORR) and oxygen evolution reaction (OER), but also the electrochemical instability during repeated charge and discharge processes [3,6,7,8]. Therefore, exploring highly efficient and robust bifunctional catalysts to accelerate sluggish ORR and OER processes and enhanced stability is still a great challenge.

Currently, the noble metals with the high catalyst activity, good conductivity, and favorable mechanical properties are used as the state-of-the-art benchmark catalysts for ORR and OER. Unfortunately, a good catalyst for ORR usually has poor activity toward OER and vice versa. For example, Pt-based catalysts exhibit excellent ORR activity, while Ir (or Ru)-based catalysts show remarkable OER activity [9,10]. Furthermore, high cost, scarcity, and poor stability terribly hinder their extensive applications in Zn-air batteries [11,12,13]. Therefore, non-noble metal based catalysts with the merits of low-cost, earth-abundant reserves, and excellent stability have attracted tremendous attention. So far, a series of great achievements have been accomplished, such as oxides [14], metal carbides [15], chalcogenides [16], non-noble metal complexes [17,18], and heteroatoms doped carbon materials [19,20]. Among them, a class of composite catalysts that were obtained by encapsulating transition metal nanoparticles in nitrogen-doped carbon (M@N-C) [21,22,23] are considered as a kind of promising candidate for ORR and OER due to the following advantages: (1) metal nanoparticles can not only facilitate the graphitization degree of external carbon shell [24], but also enhance electrons transfer to the external carbon shell [25]; (2) the nitrogen atoms that are doped in the carbon lattices can induce charge redistribution of the neighbouring carbon atoms, resulting in altering the chemisorption mode of O_2_ in form of the parallel diatomic adsorption to improve electrocatalytic activity [26,27]; and, (3) the synergetic effect that is raised from nitrogen doping and encapsulated metal nanoparticles in carbon shell heightens the inherent electrocatalytic ability on the M@N-C materials [25,28]. Although some nanocomposites of transition metal nanoparticles that are encapsulated in carbon have been successfully synthesized, it is still in the early stage of development and some shortcomings need to be overcome. For example, the low contents of transition metal and nitrogen doped in carbon matrix [29], the large size of encapsulated transition metal particles, and the too thick carbon shell result in low active site density and inefficient catalytic activity [24,30,31]. Consequently, the great challenge of M@N-C is the design and synthesis of small size transition metal nanoparticles encapsulated in thin N-doped carbon shells/layers with high loading and dispersion.

Recent studies prove that various solid phase precursor can be used to synthesize M@N-C materials by the facile pyrolysis process. This synthesis strategy has many advantages, such as simple equipment and process, high yield, and convenience of N doping in carbon matrix, making it a prospective method to the large scale commercialization. Herein, we demonstrate that cobalt nanoparticles encapsulated in N-doped carbon nanoshells (named as Co@N-C) as efficient and durable bifunctional electrocatalysts have been prepared by a convenient pyrolysis route of the solid-phase cyclen-Co-dicyandiamide complex precursor. The structure and morphology of prepared samples exhibited strong dependence on pyrolysis temperature. With an increased temperature from 700 to 900 °C, the morphology of carbon changed from carbon nanoshells to carbon nanotubes. Due to the synergistic effect of nitrogen-doping and Co encapsulating, the optimized Co@N-C-800 catalyst displayed outstanding bifunctional activities for ORR and OER in alkaline solution, which is comparable to commercial Pt/C and IrO_2_ catalyst. Moreover, the rechargeable zinc-air battery employing the optimal Co@N-C-800 catalyst as the air electrode exhibited high performance and cycling stability when compared to other counterparts.

## 2. Materials and Methods 

### 2.1. Materials

Cobaltous nitrate hexahydrate (Co(NO_3_)_2_∙6H_2_O), 1,4,7,10-tetraazacyclododecane (cyclen, C_8_H_20_N_4_), dicyandiamide (C_2_H_4_N_4_), perchloric acid (HClO_4_), potassium hydroxide, isopropanol, and ethanol are analytical grade without further purification. Distilled water was used throughout.

### 2.2. Synthesis 

Typically, 1,4,7,10-tetraazacyclododecane (172 mg) was diffused in 10 mL distilled water. Subsequently, Co(NO_3_)_2_∙6H_2_O (145 mg) was completely dissolved in 10 mL distilled water by ultrasonic and then slowly dropped into above 1,4,7,10-tetraazacyclododecane solution with constant stirring for 12 h. After that, dicyandiamide (3 g) was first dispersed in hot distilled water (60 °C) and also dropped into the above mixture solution with constantly stirring for another 6 h. After being thoroughly dried, the obtained powder was calcinated at 800 °C for 1 h (N_2_ atmosphere, ramp rate: 5 °C min^−1^, flowing rate: 50 cc min^−1^). To remove unstable cobalt species, the powder was then immersed in 80 °C HClO_4_ solution (0.5 M, 100 mL) for 8 h. Finally, the black powder was thoroughly cleaned with distilled water and ethanol, dried at 60 °C in vacuum, and marked as Co@N-C-800, in which “800” represents the pyrolysis temperature. To study the influence of different pyrolysis temperature on the morphology and electrochemical performance of Co@N-C hybrid catalysts, the Co@N-C-700 and Co@N-C-900 samples were also synthesized by the same process.

### 2.3. Characterizations

The products were characterized by a Rigaku/Max-3A diffractometer with Co Ka radiation (XRD, Malvern Panalytical, Almelo, The Netherlands), a scanning electron microscope (SEM, Supra 55 Sapphire, HITACHI, Tokyo, Japan) equipped with an energy dispersive X-ray spectrometer (EDX, Hitachi Limited, Tokyo, Japan), a transmission electron microscope (TEM; JEOL-2100, JEOL, Tokyo, Japan), and an X-ray photoelectron spectrometer (XPS, Thermo ESCALAB 250XI, Thermo Fisher Scientific Inc., Waltham, MA, USA). The thermo gravimetric (TG) was operated on a Thermo Gravimetric Analyzer (TA Instruments Q500, TA Instruments, New Castle, PA, USA) from 25 °C to 800 °C in air (heating rate: 10 °C min^−1^).

### 2.4. Electrochemical Measurements

All electrochemical measurements were recorded by an electrochemical workstation (CHI 660E, CH Instruments Inc., Shanghai, China) connected with a rotating disk electrode (RDE) in a three-electrode system with O_2_- or N_2_-saturated 0.1 M KOH solution, where Pt wire, glassy carbon electrode (GCE, 3 mm in diameter), and silver-silver chloride electrode (Ag/AgCl) were served as the counter electrode, working electrode, and reference electrode, respectively. The potentials were normalized to the reversible hydrogen electrode (RHE) by using the following equation [32]:

*E*_RHE_ = *E*_Ag/AgCl_ + 0.059 pH + 0.197 (1)

The fabrication of working electrode: 2.4 mg of obtained catalyst was added in a mixture solution, including distilled water (735 μL), isopropanol (205 μL), and 5 wt % Nafion (60 μL, Alfa, Shanghai, China) by ultrasound to form a homogeneous ink. After that, 3 μL of the above catalyst ink was dropped onto the GC electrode and naturally dried at room temperature. The loading was 100 μg cm^−2^. The commercial Pt/C (20 wt %, Johnson Matthey, Shanghai, China) and IrO_2_ (Alfa, Shanghai, China) catalysts with the same loading were used as the benchmark for ORR and OER, respectively.

For the ORR test, the static cyclic voltammogram (CV) was operated between 0.1 and 1.0 V (vs. RHE) at the scan rate of 50 mV s^−1^. The linear sweep voltammograms (LSV) was executed from 1.0 V to 0.1 V (vs. RHE) at 10 mV s^−1^ with a series of rotation speed from 600 to 2000 rpm. Koutecky-Levich (K-L) plots were estimated at diverse potentials (0.1–0.4 V vs. RHE). The transfer electron number (*n*) was calculated by the following K-L formula:

*j*^−1^ = *j*_L_^−1^ + *j*_K_^−1^ = (*Bω*^1/2^)^−1^ + *j*_K_^−1^ (2)

*B* = 0.2*nFC_o_D_o_*^2/3^ν^−1/6^ (3)

*j*_K_ = *nFkC*_o_ (4)

where *j*, *j*_L_, *j*_K_, and *ω* are the measured current density, diffusion-limiting current density, kinetic-limiting current density, and angular velocity, respectively. *n*, *F*, *C*_o_, *D*_o_, ν, and *k* are the transferred electron number of the ORR, the Faraday constant, the bulk concentration of O_2_, the diffusion coefficient of O_2_, the kinematic viscosity of the electrolyte, and the electron-transfer rate constant, respectively.

For the OER test, the LSVs were tested from 1.0 V to 1.86 V (vs. RHE) in O_2_-saturated electrolyte (10 mV s^−1^, rotating speed: 1600 rpm). All polarization curves were corrected with 95% iR-compensation.

### 2.5. Air Electrodes Preparation 

The Vulcan XC-72R (CABOT) and Polyvinylidene Fluoride (PVDF) with a mass ratio of 8:1 was mixed and coated onto the carbon paper (Toray TGP-H-060, TORAY, Tokyo, Japan) to obtain the air diffusion layer with the loading of ~2 mg cm^−2^. 9 mg of catalyst was dispersed in the solution of isopropanol (900 μL) and 5 wt % nafion solution (100 μL) by ultrasonic to form a homogeneous ink. Subsequently, the catalyst ink (100 μL) was dropped on the above air diffusion layer and slowly dried in vacuum at an ambient temperature. The commercial Pt/C based air electrode was also prepared under the same method. The loading of the catalyst was all about 1 mg cm^−2^.

### 2.6. Zn-Air Battery Test

The performances of zinc-air battery were investigated on a home-made cell. Zinc plate, prepared air electrode, and 6 M KOH with 0.2 M zinc acetate solution were used as the anode, cathode, and electrolyte, respectively. Polarization and galvanostatic charge/discharge measurements were performed on the CHI 660E electrochemical station and LAND testing system, respectively. All of the tests were provided continuous oxygen from ambient air.

## 3. Results and Discussion

### 3.1. Structural Characterization

**Figure 1:**
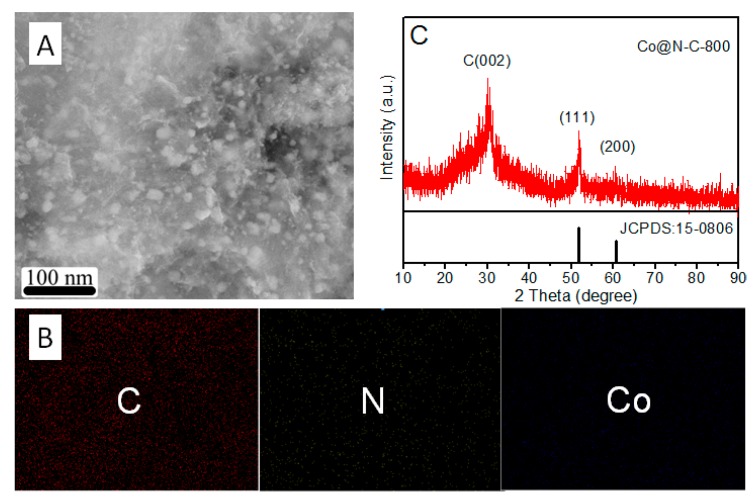
(**A**) scanning electron microscope (SEM) images of Co@N-C-800, (**B**) the element mapping of Co@N-C-800 corresponding to image a, (**C**) X-ray diffractometer (XRD) pattern of Co@N-C-800.

**Figure 2:**
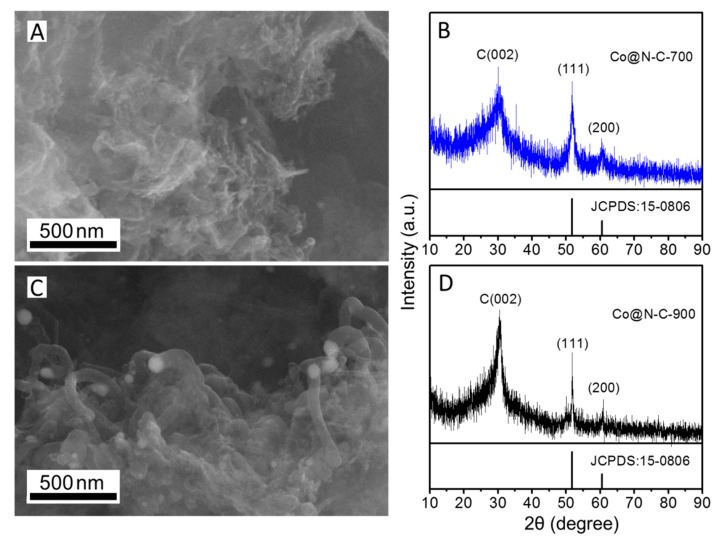
(**A**) SEM image and (**B**) XRD pattern of Co@N-C-700, (**C**) SEM image, and (**D**) XRD pattern of Co@N-C-900.

The Co@N-C-800 sample was first studied by the scanning electron microscope and X-ray diffraction. SEM image (Figure 1A) clearly shows that cobalt nanoparticles (~20 nm) are well distributed in carbon materials. The element mapping images (Figure 1B) indicate the homogeneous dispersion of cobalt and nitrogen elements in the carbon matrix. The two obvious diffraction peaks that were located at 51.8° and 60.6° in the XRD pattern (Figure 1C) can be well matched with (111) and (200) lattice planes of cubic phase of Co (JCPDS:15-0806). Furthermore, the diffraction peak at about 26° belong to C (002), also shown in the XRD pattern, indicating the successful formation of carbonaceous materials by the carbonization of cyclen [33]. No impurity peaks attributed to cobalt oxides are detected, implying that the precursor was well reduced to Co nanoparticles and then protected by the exterior carbon nanoshells and N_2_ atmosphere. For comparison, Co@N-C-700 and Co@N-C-900 show similar XRD patterns but different morphologies, indicating that the temperature is the key factor to tune the morphology of catalysts (Figure 2). 

**Figure 3:**
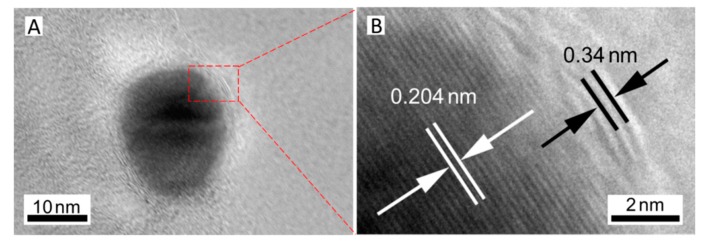
(**A**) transmission electron microscope (TEM) image and (**B**) high resolution transmission electron microscope (HRTEM) image of Co@N-C-800.

**Figure 4:**
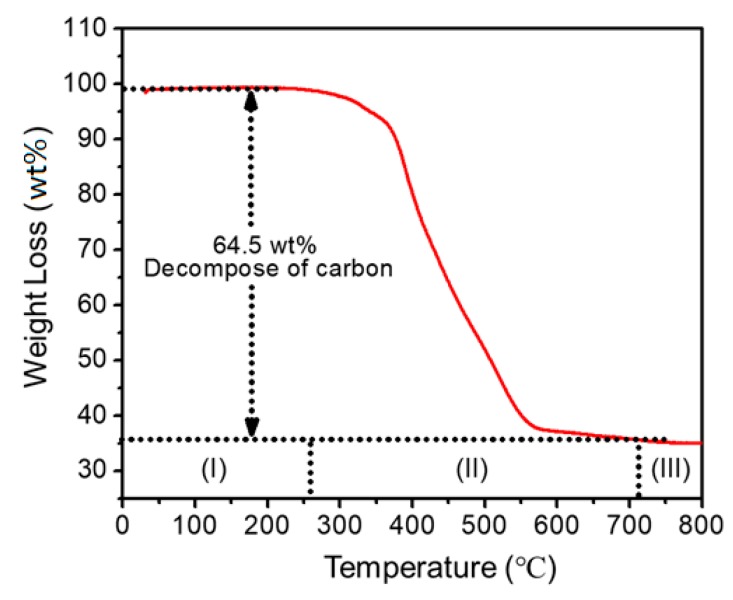
Thermogravimetric Analysis (TGA) curve of Co@N-C-800.

TEM images are also displayed to discuss the structural characterization, as presented in Figure 3A, which shows that the Co nanoparticles are tightly encapsulated with very thin graphene-like carbon nanoshells (less than five layers). The lattice fringe spaces of 0.203 nm and 0.34 nm revealed in HRTEM image (Figure 3B) are assigned to the (111) plane of Co nanoparticles and the (002) plane of graphitic carbon, respectively, which are well consistent with the XRD. It needs to be emphasized that this novel nanostructure of monodispersed Co nanoparticles tightly wrapped with very thin graphene-like carbon nanoshells can not only effectively protect and avoid the oxidation of Co nanoparticles, but also enhance the catalytic activity, owing to the high loading and dispersion of cobalt nanoparticles. Refs. [24,31,34] The high density of Co nanoparticles in Co@N-C-800 was examined by TG analysis (Figure 4). According to the TG result, the obtained Co@N-C-800 powder was very dry and only 0.5% weight lost was detected, owing to the residual H_2_O. After the temperature was raised to 300 °C, a fast weight drop was recorded, which can be attributed to the decomposition of carbon materials and 35% of weight was finally remained when the temperature reached 800 °C. The Co have been oxidized and changed to Co_3_O_4_ during the heating process under the air condition, so the exact Co content of the Co@N-C-800 is calculated to be about 25.8%.

**Figure 5:**
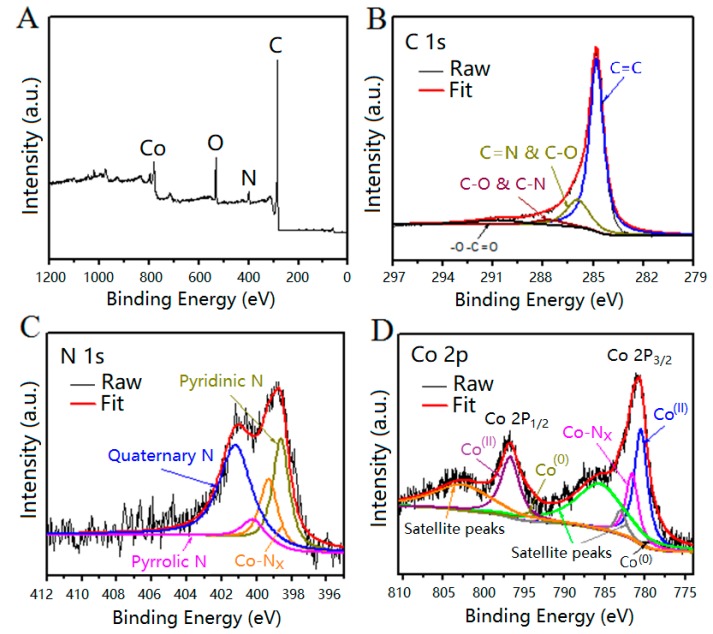
X-ray photoelectron spectrometer (XPS) spectrum of Co@N-C-800. (**A**) Survey spectra; the high resolution spectrum of (**B**) C 1s, (**C**) N 1s, and (**D**) Co 2p.

The chemical states and surface composition of the Co@N-C-800 were investigated by XPS. As provided in Figure 5A, a series of peaks attribute to C 1s, Co 2p, O 1s, and N 1s were detected in the survey spectrum, which further confirm the existence of C, Co, and N elements in the Co@N-C-800 catalyst and N-doping reaction do occur in the carbon shell during the preparation process. The high-resolution C 1s displays four types of C species, including C=C (284.6 eV), C=N (285.9 eV), C–N&C–O (287.6 eV), and O=C–O– (290.6 eV), [35,36] indicating the doping of heteroatoms (N and O) in the carbon framework (Figure 5B). The high-resolution N 1s spectrum that is presented in Figure 5C is fitted to four peaks corresponding to pyridinic-N (398.5 eV), metal-N (399.3 eV), pyrrolic-N (400.2 eV), and quaternary-N (401.5 eV), respectively. Refs. [37,38] Moreover, the pyridinic-N, metal-N, and quaternary-N take the dominant position of N species, which is beneficial for oxygen electrocatalysis by providing more active sites and improving the electron transfer of the carbon matrix [36,37,38,39]. The Co 2p_1/2_ and Co 2p_3/2_ high-resolution spectrum (Figure 5D) can be fitted with three components corresponding to Co (0) (778.6 and 794.3 eV), Co (II) (780.4 and 796.7 eV), and the satellite peaks (782.6, 785.2, and 802.5 eV). Refs. [36,40] Moreover, the peak at 781.6 eV in Co 2p_3/2_ spectra indicates the possible presence of Co-N*_x_*, which are also active sites for oxygen electrocatalysis and match well with the metal-N peak (399.3 eV) in N 1s spectrum [38].

### 3.2. The Formation Mechanism of Co-N/C-800

**Scheme 1:**
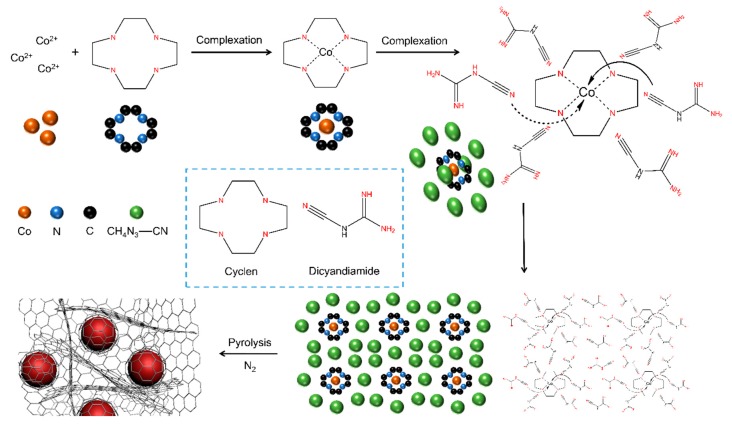
Illustration of synthetic procedure and the formation mechanism for Co@N-C-800.

As a common macrocyclic ligand, 1,4,7,10-tetraazacyclododecane (also called as cyclen) was chosen to be the complexing agent in our experiment due to its strong and selective binding ability to metal cations and high nitrogen content in molecule. The synthetic procedure and possible formation mechanism of Co@N-C catalysts could be described by Scheme 1. Firstly, when be dissolved into water together with Co^2+^ ion under constantly stirring, the unique cavity of the cyclen molecule can bind and anchor the Co^2+^ ion by the four-NH groups occupying the partial coordination sites of Co^2+^ ion to form Co-cyclen complex. After the dicyandiamide solution was introduced to the above reaction system, the –CN group derived from dicyandiamide can coordinate with Co-cyclen complex by occupying the residual sites of Co^2+^ ion to form the cyclen-Co-dicyandiamide complex subsequently. Furthermore, the dicyandiamide molecules can link with each other by hydrogen bonds between –NH_2_ (or =NH) groups and –NH– groups to form a network on which the Co-cyclen complex molecules were uniformly distributed and anchored. Finally, after pyrolyzed at high temperature in N_2_, the cyclen molecule was transformed to nitrogen-doped thin carbon nanoshells to encapsulate Co nanoparticles, resulting in the formation of Co@N-C hybrid catalyst. More importantly, the dicyandiamide molecule units also transformed into N-doped graphene-like carbon layers during the pyrolysis process [35,41] and surrounded the Co@N-C to provide a continuous and complete conductive network. The unique nanostructure of Co nanoparticles encapsulated in thin N-doped carbon nanoshells and uniformly distributed in graphene-like carbon layers can provide high-density catalytic active sites and continuous and complete electron transfer path at the same time, resulting in excellent bifunctional activity to ORR and OER and high performance when used as a catalytic electrode for rechargeable Zn-air battery.

### 3.3. Electrochemical Study

**Figure 6:**
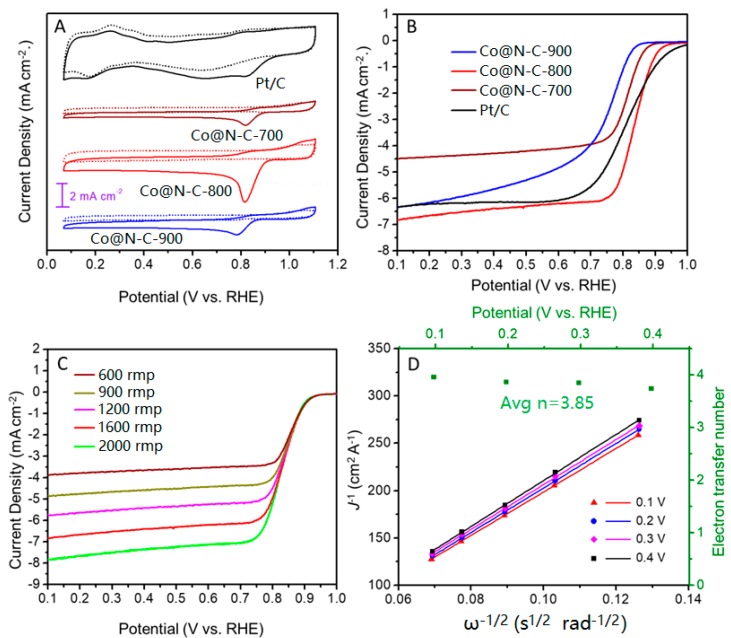

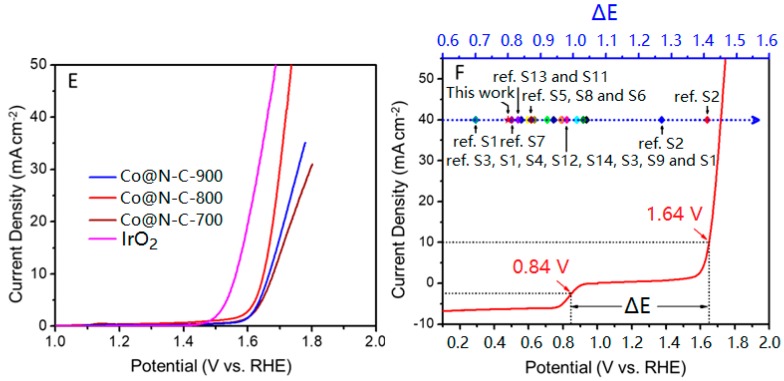
ORR and oxygen evolution reaction (OER) performances of Co@N-C and Pt/C with the loading of 100 μg cm^−2^ using O_2_-saturated 0.1 M KOH as electrolyte. (**A**) Static cyclic voltammogram (CV) curves of different catalysts (solid line: in O_2_-saturated electrolyte; dotted line: in N_2_-saturated electrolyte), (**B**) linear sweep voltammograms (LSV) plots of catalysts for ORR at 1600 rmp, (**C**) ORR polarization curves of Co@N-C-800 at different rotating speeds (600 to 2000 rpm), (**D**) the K-L plots of Co@N-C-800 derived from rotating disk electrode (RDE) data and electron transfer number (*n*) per oxygen molecule (O_2_) at potentials from 0.1 to 0.4 V, (**E**) LSV plots of catalysts for OER at 1600 rmp and (**F**) the bifunctional catalytic activities of Co@N-C-800 within the ORR and OER potential window.

The electrochemical properties were studied by a three-electrode system using 0.1 M KOH as electrolyte. The loading of electrocatalyst on the working electrode was same for all. The ORR catalytic activity of different catalysts was firstly evaluated by static CV tests in O_2_-saturated and N_2_-saturated electrolyte, respectively. As given in Figure 6A, the catalytic activity of Co@N-C-800 catalyst is higher than all of the other prepared electrocatalysts. The onset potential (0.937 V) and cathodic peak potential (0.818 V) of Co@N-C-800 were just 19 mV and 3 mV more negative than the benchmark Pt/C, respectively. Moreover, the Co@N-C-800 catalyst reached an astonishing cathodic peak current of 3.80 mA cm^−2^, which was almost three times than that of commercial Pt/C (1.37 mA cm^−2^) (Table 1). The ORR activity of the various catalysts is further studied by RDE. According to the linear sweep voltammetry (LSV) curves that were obtained at a scan rate of 10 mV s^−1^ and rotating rate of 1600 rpm, the Co@N-C-800 catalyst also presents the highest ORR activity among all the prepared Co@N-C catalysts and it is comparable to commercial Pt/C. Although the onset potential of Co@N-C-800 is slightly lower than that of Pt/C, the half-wave potential (*E*_1/2_), which indicates how well the system performs under a specific current, is even 29 mV more positive than Pt/C catalyst (0.842 V vs. 0.813 V), and the limiting current density of Co-N/C-800 (6.49 mA cm^−2^) is also higher than the Pt/C catalyst (6.14 mA cm^−2^) (Figure 6B). In order to explore the ORR kinetics of Co@N-C-800, the ORR polarization curves at different rotating rates from 600 to 2000 rmp were also collected. As shown in Figure 6C, the polarization curves at different rotating speeds all clearly show three areas, including (1) kinetic controllable area, (2) mixed kinetic-diffused controllable area, and (3) diffused controllable area. According to the above K-L formula, the K-L curves of Co@N-C-800 catalyst are obtained by the linear fit of reciprocal of current density (*j*^−1^) and the reciprocal of square root of angular velocity (*ω*^−1/2^) at various potentials. The electron transferred number (*n*) is calculated to be 3.85 (Figure 6D), implying a predominant four electron ORR pathway, corresponding to the reaction of O_2_ + 2H_2_O + 4e^−^ → 4OH^−^. The Co@N-C-800 also possesses the highest OER activity among all of the prepared Co@N-C catalysts and it is only outperformed by the state-of-the-art IrO_2_ catalyst (Figure 6E). The bifunctional catalytic activity of oxygen catalyst can be evaluated by the difference in potential (Δ*E*) between ORR process at the current density of 3 mA cm^−2^ and OER process at the current density of 10 mA cm^−2^. Refs. [22,36] Usually, a catalyst with a lower Δ*E* value will exhibit good bifunctionality for ORR and OER. The low Δ*E* value of 0.80 V for Co@N-C-800 (Figure 6F) is smaller than many previous reported catalysts (Table S1), indicating the better bifunctional catalytic activity for both ORR and OER.

### 3.4. Rechargeable Zn-Air Batteries

**Figure 7:**
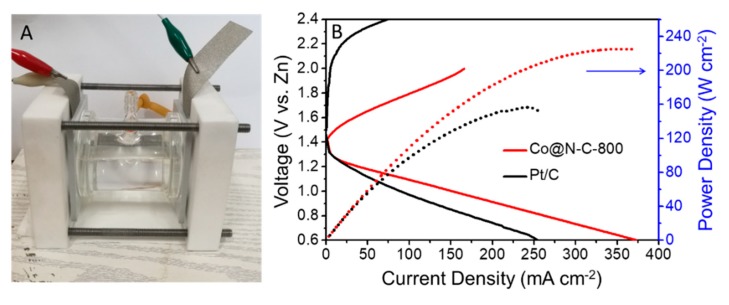

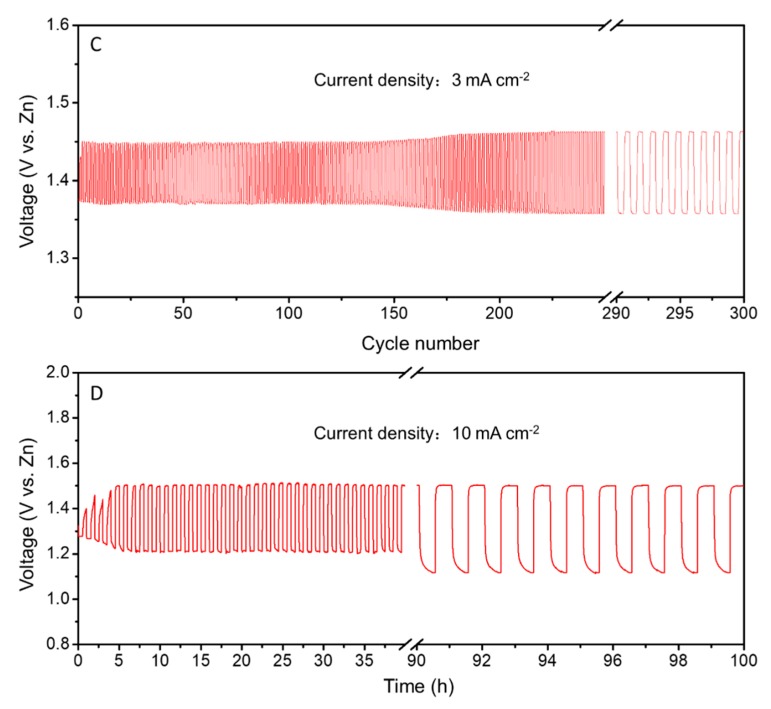
The performances of Zn-air battery with Co@N-C-800 or Pt/C as air cathode. (**A**) The illustration of the home-made Zn-air battery device, (**B**) charge/discharge polarization profiles and power density curves of the batteries, (**C**) a fast charge-discharge cycling test of the battery using Co@N-C-800 catalyst at current density of 3 mA cm^−2^ in a total of 300 cycles, and (**D**) a long-term change-discharge test of the batteries with Co@N-C-800 catalyst in 100 h cycle period at 10 mA cm^−2^ (1 h per cycle).

Apart from the above traditional electrochemical studies, a home-made rechargeable Zn-air battery was used to further evaluate the performance of Co@N-C-800 catalyst as the air cathode material under real battery operation conditions (Figure 7A). The Pt/C based electrode was also prepared and tested with the same manner. The charge and discharge polarization and power density curves of batteries based on Co@N-C-800 and Pt/C catalyst are shown in Figure 7B. It can be clearly found that the battery performance of Co@N-C-800 cathode is superior to the Pt/C cathode. The zinc–air battery based on Co@N-C-800 cathode achieves a high open circuit voltage of 1.45 V and the charge–discharge voltage gap of Co-N/C-800 is much smaller than that of Pt/C. During the discharge procedure, the Co@N-C-800 based battery can reach a high current density of 157 mA cm^−2^ at 1.0 V, while the Pt/C based battery only reach a current density of 120 mA cm^−2^ at the same voltage. During the charge process, Co@N-C-800 based Zn-air battery also demonstrates a better performance than Pt/C based battery. Moreover, the maximum peak power density of the Zn-air battery based on Co@N-C-800 cathode achieves 255 mW cm^−2^, which is far greater than Pt/C catalyst (156 mW cm^−2^). The cycle stability of the rechargeable Zn-air battery based on Co@N-C-800 catalyst was investigated and characterized by the fluctuation of the charge potential (*E*_CP_) and discharge potential (*E*_DP_) [42]. A fast charge-discharge cycling test was firstly performed at the current density of 3 mA cm^−2^ and is shown in Figure 7C (one cycle includes 5 min discharge and 5 min charge). It is obviously shown that the Co@N-C-800 based battery exhibited great stability with the *E*_CP_ increased only 0.9% and *E*_DP_ decayed 0.8% in total 300 cycles. After that, long-term charge-discharge cycling durability (a cycle includes 30 min discharge followed by 30 min charge) was further investigated at a higher current density of 10 mA cm^−2^ (Figure 7D). Obviously, the excellent long-term stability of Co-N/C-800 based battery is proved by almost no rise for *E*_CP_ and limited voltage drop for *E*_DP_ (7.4% decay) in total 100 h cycle period. All of the above results demonstrate the outstanding performances of Co@N-C-800 catalyst in rechargeable Zn-air battery are comparable or even superior to the state-of-the-art Pt/C catalyst and many previously reported air cathodes (Table S2). The superior bifunctional electrocatalytic activitiy and outstanding performance in rechargeable Zn-air battery for Co@N-C-800 catalyst are probably contribute to the synergetic effect of nanocomposite catalyst: (1) Co nanoparticles encapsulated with thin nitrogen-doped carbon nanoshells and uniformly distributed in graphene-like carbon layers can not only avoid agglomeration to provide high-density catalytic active sites, but also improve the stability and electrical conductivity; and, (2) the N doping can induce the change of charge distribution and form high density of defects, which also can be employed as active sites for OER and ORR. Although the in-depth mechanism needs to be further studied, the excellent electrocatalytic properties together with ambient air rather than pure O_2_ and very small loading of 1 mg make Co@N-C-800 catalyst to be a promising candidate for practical application in rechargeable Zn-air battery.

## 4. Conclusions

In summary, a novel nanocomposite of high-density cobalt nanoparticles encapsulated in very thin N-doped carbon nanoshells was successfully synthesized by simple thermal decomposition of cyclen-Co-dicyandiamide solid-phase precursor. The morphologies and properties of products can be easily tuned by changing the pyrolysis temperature. Due to the synergetic effect of nanocomposite, the optimized Co@N-C-800 catalyst showed outstanding bifunctional catalytic activities for both ORR and OER in alkaline electrolyte. Furthermore, the rechargeable Zn-air battery that is based on Co@N-C-800 air cathode presented excellent electrochemical performance and strong cycle stability. The work demonstrated here provides a new strategy and approach to design and prepare advanced nanocomposites of transition metal nanoparticles and N-doped carbon materials for highly efficient bifunctional electrocatalysts in the high-performance rechargeable Zn-air battery and the other catalytic field.

## Figures and Tables

**Table 1:** Catalytic activity data of various catalysts for oxygen reduction reaction (ORR).

Electrocatalyst	Onset Potential (V vs. RHE)	Cathodic Peak Potential (V vs. RHE)	Cathodic Peak Current Density (mA cm^−2^)
Pt/C	0.956	0.821	1.37
C@Co-700	0.919	0.818	1.18
C@Co-800	0.937	0.818	3.80
C@Co-900	0.881	0.783	1.33

## Supplementary Materials

The following are available online at http://www.mdpi.com/1996-1944/12/2/243/s1, Table S1: Comparison of bifunctional oxygen electrode activity data for different catalysts; Table S2: Comparison of the performances of rechargeable Zn–air battery for different catalysts.
