# Supplementary material for: High-Density Cobalt Nanoparticles Encapsulated with Nitrogen-Doped Carbon Nanoshells as a Bifunctional Catalyst for Rechargeable Zinc-Air Battery
Source: Materials (Basel). 2019 Jan 12;12(2):243. doi: 10.3390/ma12020243 (PMC6356503; doi:10.3390/ma12020243)
Supplement: Supplementary file 1 [file materials-12-00243-s001.pdf]

materials

Supplementary Materials

High-Density Cobalt Nanoparticles Encapsulated with Nitrogen-Doped Carbon Nanoshells as a Bifunctional Catalyst for Rechargeable Zinc-Air Battery

Shuqi Liang and Ce Liang *

Key Laboratory of Automobile Materials, Ministry of Education, and College of Materials Science and Engineering, Jilin University, Changchun 130025, China; lsqjlu@163.com
* Correspondence: liangce@jlu.edu.cn; Tel.: +86-431-8509-5170

Table 1. Comparison of bifunctional oxygen electrode activity data for different catalysts.

Catalysts	Loading ($\mu g\cdot cm^{-2}$)	E_{ORR}/V ($I = -3$ mA·cm^{-2})	E_{OER}/V ($I = 10$ mA·cm^{-2})	ΔE/V ($E_{OER}-E_{ORR}$)	Electrolyte	Source
Pt/C	200	0.82	1.86	1.04	0.1 M KOH	ref. [1]
IrO_2	210	0.29	1.70	1.41	0.1 M KOH	ref. [2]
20% Ir/C	28	0.69	1.61	0.92	0.1 M KOH	ref. [3]
RuO_2	210	0.37	1.64	1.27	0.1 M KOH	ref. [2]
20% Ru/C	28	0.61	1.62	1.01	0.1 M KOH	ref. [3]
Co-N/C 800	240	0.78	1.74	0.96	0.1 M KOH	ref. [4]
Co/N-C-800	250	0.74	1.60	0.86	0.1 M KOH	ref. [5]
Fe@N-C	~311	0.83	1.71	0.88	0.1 M KOH	ref. [6]
Fe/N-CNTs	200	0.81	1.75	0.94	0.1 M KOH	ref. [1]
CNCN-44	200	0.80	1.61	0.81	0.1 M KOH	ref. [7]
(Ni,Co)/CNT	240	0.74	1.60	0.87	0.1 M KOH	ref. [8]
meso-Co_3O_4-35	100	0.61	1.64	1.03	0.1 M KOH	ref. [9]
FeCo@NC-750	800	~0.79	1.49	0.70	0.1 M KOH	ref. [10]
Ni_3Fe/N-C	130	0.78	1.62	0.84	0.1 M KOH	ref. [11]
N-graphene/CNT	~424	0.69	1.65	0.96	0.1 M KOH	ref. [12]
m-NiFe/CNx	200	0.76	1.59	0.83	0.1 M KOH	ref. [13]
$CoFe_2O_4$/rGO	1006	0.73	1.71	0.98	0.1 M KOH	ref. [14]
Co-N/C-800	100	0.84	1.64	0.80	0.1 M KOH	this work

Table 2. Comparison of the performances of rechargeable Zn–air battery for different catalysts.

Catalysts	Loading (mg·cm^{-2})	Per Cycle (min)	Cycle Time (h)	Fluctuation of Discharge Potential (E_{DP}) (V, initial/end)	Fluctuation of Charge Potential (E_{CP}) (V, initial/end)	Current Density (mA·cm^{-2})	Source
NCNT/CoO-NiO-NiCo	0.53	10	17	~ 1.11/1.06	~2.03/1.97	20	ref. [15]
Fe@N-C	2.2	10	16.7	1.25/1.11	1.95/1.97	10	ref. [6]
Co-PDA-C	1	60	500	1.21/1.01	2.15/2.18	2	ref. [16]
$CoMn_2O_4$/N-rGO	-	10	~16.7	~1.09/1.01	~1.77/1.89	20	ref. [17]
Ni_3Fe/N-C sheets	-	240	420	~1.17/1.05	~1.98/2.06	10	ref. [11]
FeCo@NC-750	1	10	20	~1.21/1.14	~1.96/2.00	10	ref. [10]
$NiCo_2O_4$-CNTs	2	10	40	~1.30/1.22	~2.08/2.17	10	ref. [18]
Co-N/C-800	1	60	100	1.21/1.12	1.50/1.51	10	This work

Reference

1. Y. Liu, H. Jiang, Y. Zhu, X. Yang, C. Li, Transition metals (Fe, Co, and Ni) encapsulated in nitrogen-doped carbon nanotubes as bi-functional catalysts for oxygen electrode reactions, J. Mater. Chem. A., 4 (2016) 1694–1701.
2. A. Aijaz, J. Masa, C. Rösler, W. Xia, P. Weide, A.J.R. Botz, R.A. Fischer, W. Schuhmann, M. Muhler, Co@Co3O4 Encapsulated in Carbon Nanotube-Grafted Nitrogen-Doped Carbon Polyhedra as an Advanced Bifunctional Oxygen Electrode, Angew. Chemie Int. Ed., 55 (2016) 4087–4091.
3. Y. Gorlin, T.F. Jaramillo, A bifunctional nonprecious metal catalyst for oxygen reduction and water oxidation, J. Am. Chem. Soc., 132 (2010) 13612–13614.
4. W. Hu, Q. Wang, S. Wu, Y. Huang, Facile one-pot synthesis of a nitrogen-doped mesoporous carbon architecture with cobalt oxides encapsulated in graphitic layers as a robust bicatalyst for oxygen reduction and evolution reactions, J. Mater. Chem. A., 4 (2016) 16920–16927.
5. Y. Su, Y. Zhu, H. Jiang, J. Shen, X. Yang, W. Zou, J. Chen, C. Li, Cobalt nanoparticles embedded in N-doped carbon as an efficient bifunctional electrocatalyst for oxygen reduction and evolution reactions, Nanoscale, 6 (2014) 15080–15089.
6. J. Wang, H. Wu, D. Gao, S. Miao, G. Wang, X. Bao, High-density iron nanoparticles encapsulated within nitrogen-doped carbon nanoshell as efficient oxygen electrocatalyst for zinc–air battery, Nano Energy, 13 (2015) 387–396.
7. J. Song, C. Zhu, S. Fu, Y. Song, D. Du, Y. Lin, Optimization of cobalt/nitrogen embedded carbon nanotubes as an efficient bifunctional oxygen electrode for rechargeable zinc–air batteries, J. Mater. Chem. A., 4 (2016) 4864–4870.
8. N. Ma, Y. (Alec) Jia, X. Yang, X. She, L. Zhang, Z. Peng, X. Yao, D. Yang, Seaweed biomass derived (Ni,Co)/CNT nanoaerogels: efficient bifunctional electrocatalysts for oxygen evolution and reduction reactions, J. Mater. Chem. A., 4 (2016) 6376–6384.
9. Y.J. Sa, K. Kwon, J.Y. Cheon, F. Kleitz, S.H. Joo, Ordered mesoporous Co3O4 spinels as stable, bifunctional, noble metal-free oxygen electrocatalysts, J. Mater. Chem. A., 1 (2013) 9992.
10. P. Cai, S. Ci, E. Zhang, P. Shao, C. Cao, Z. Wen, FeCo Alloy Nanoparticles Confined in Carbon Layers as High-activity and Robust Cathode Catalyst for Zn-Air Battery, Electrochim. Acta, 220 (2016) 354–362.
11. G. Fu, Z. Cui, Y. Chen, Y. Li, Y. Tang, J.B. Goodenough, Ni3Fe-N Doped Carbon Sheets as a Bifunctional Electrocatalyst for Air Cathodes, Adv. Energy Mater., 7 (2017) 1–8.
12. Z. Wen, S. Ci, Y. Hou, J. Chen, Facile one-pot, one-step synthesis of a carbon nanoarchitecture for an advanced multifunctonal electrocatalyst, Angew. Chemie. Int. Ed., 53 (2014) 6496–6500.
13. S. Ci, S. Mao, Y. Hou, S. Cui, H. Kim, R. Ren, Z. Wen, J. Chen, Rational design of mesoporous NiFe-alloy-based hybrids for oxygen conversion electrocatalysis, J. Mater. Chem. A., 3 (2015) 7986–7993.
14. W. Bian, Z. Yang, P. Strasser, R. Yang, A CoFe2O4/graphene nanohybrid as an efficient bi-functional electrocatalyst for oxygen reduction and oxygen evolution, J. Power Sources, 250 (2014) 196–203.

15. X. Liu, M. Park, M.G. Kim, S. Gupta, G. Wu, J. Cho, Integrating NiCo Alloys with Their Oxides as Efficient Bifunctional Cathode Catalysts for Rechargeable Zinc-Air Batteries, Angew. Chemie. Int. Ed., 54 (2015) 9654–9658.
16. B. Li, Y. Chen, X. Ge, J. Chai, X. Zhang, T.S.A. Hor, G. Du, Z. Liu, H. Zhang, Y. Zong, Mussel-inspired one-pot synthesis of transition metal and nitrogen co-doped carbon (M/N–C) as efficient oxygen catalysts for Zn-air batteries, Nanoscale, 8 (2016) 5067–5075.
17. M. Prabu, P. Ramakrishnan, S. Shanmugam, CoMn2O4 nanoparticles anchored on nitrogen-doped graphene nanosheets as bifunctional electrocatalyst for rechargeable zinc–air battery, Electrochem. Commun., 41 (2014) 59–63.
18. C. Ma, N. Xu, J. Qiao, S. Jian, J. Zhang, Facile synthesis of NiCo2O4 nanosphere-carbon nanotubes hybrid as an efficient bifunctional electrocatalyst for rechargeable Zn-air batteries, Int. J. Hydrogen Energy, 41 (2016) 9211–9218.
